# Genomic analysis of nontypeable pneumococci causing invasive pneumococcal disease in South Africa, 2003–2013

**DOI:** 10.1186/s12864-016-2808-x

**Published:** 2016-06-22

**Authors:** Thabo Mohale, Nicole Wolter, Mushal Allam, Kedibone Ndlangisa, Penny Crowther-Gibson, Mignon du Plessis, Anne von Gottberg

**Affiliations:** Centre for Respiratory Diseases and Meningitis (CRDM), National Institute for Communicable Diseases (NICD), National Health Laboratory Services (NHLS), Johannesburg, South Africa; School of Pathology, Faculty of Health Sciences, University of the Witwatersrand, Johannesburg, South Africa; Public Health Surveillance and Response, National Institute for Communicable Diseases, National Health Laboratory Services, Johannesburg, South Africa

**Keywords:** South Africa, *Streptococcus pneumoniae*, Nontypeable, Invasive pneumococcal disease, Whole genome sequencing

## Abstract

**Background:**

The capsular polysaccharide is the principal virulence factor of *Streptococcus pneumoniae* and a target for current pneumococcal vaccines. However, some pathogenic pneumococci are serologically nontypeable [nontypeable pneumococci (NTPn)]. Due to their relative rarity, NTPn are poorly characterized, and, as such, limited data exist which describe these organisms. We aimed to describe disease and genotypically characterize NTPn causing invasive pneumococcal disease in South Africa.

**Results:**

Isolates were detected through national, laboratory-based surveillance for invasive pneumococcal disease in South Africa and characterized by whole genome analysis. We predicted ancestral serotypes (serotypes from which NTPn may have originated) for Group I NTPn using multilocus sequence typing and capsular region sequence analyses. Antimicrobial resistance patterns and mutations potentially causing nontypeability were identified. From 2003–2013, 39 (0.1 %, 39/32,824) NTPn were reported. Twenty-two (56 %) had partial capsular genes (Group I) and 17 (44 %) had complete capsular deletion of which 15 had replacement by other genes (Group II). Seventy-nine percent (31/39) of our NTPn isolates were derived from encapsulated *S. pneumoniae*. Ancestral serotypes 1 (27 %, 6/22) and 8 (14 %, 3/22) were most prevalent, and 59 % (13/22) of ancestral serotypes were serotypes included in the 13-valent pneumococcal conjugate vaccine. We identified a variety of mutations within the capsular region of Group I NTPn, some of which may be responsible for the nontypeable phenotype. Nonsusceptibility to tetracycline and erythromycin was higher in NTPn than encapsulated *S. pneumoniae*.

**Conclusions:**

NTPn are currently a rare cause of invasive pneumococcal disease in South Africa and represent a genetically diverse collection of isolates.

**Electronic supplementary material:**

The online version of this article (doi:10.1186/s12864-016-2808-x) contains supplementary material, which is available to authorized users.

## Background

*Streptococcus pneumoniae* frequently colonizes the nasopharynx asymptomatically and is also a significant human pathogen causing diseases such as otitis media, pneumonia and meningitis [1]. The capsule is a major virulence factor of *S. pneumoniae*, protecting it from host cell-mediated phagocytosis [2]. The capsule induces protective antibodies which provide protection against pneumococcal disease, and serves as the basis for current pneumococcal vaccines [3]. Based on the structure and antigenicity of the capsule, more than 90 serotypes have been identified [4, 5].

Nontypeable pneumococci (NTPn) are not assigned a serotype when using the Quellung reaction [6]. The inability to do so may be due to low-level capsule expression or novel capsule types not detectable by the Quellung reaction, or absence of capsule due to genetic modifications in the capsular polysaccharide synthesis locus (*cps*) [7–11]. NTPn are predominantly detected in carriage or non-invasive disease episodes, and rarely cause invasive pneumococcal disease (IPD) [12–14]. In vitro studies have shown that NTPn display increased adherence to epithelial cells and are more easily transformable compared to encapsulated *S. pneumoniae* (Ec-*Sp*) isolates [15, 16].

NTPn have been categorized into two groups based on the contents of the *cps* locus [7]. Group I has at least partial *cps* genes, whereas in Group II the *cps* genes are completely deleted and may be replaced with other genes. Group II is further subdivided into four null capsule clades (NCC) [17]. NCC1 has the *psk* gene which has been shown to play a role in adherence to epithelial cells and colonisation [18]. NCC2 has *aliC* and *aliD* genes either with (NCC2a) or without (NCC2b) a putative toxin-antitoxin system (encoded by *ntaAB* genes). *AliC* and *aliD* genes facilitate upregulation of competence for genetic transformation and mediate colonization, respectively [19]. NCC3 has the *aliD* gene, and NCC4 contains only transposable elements in the *cps* locus.

There are limited studies describing invasive NTPn [10, 12], possibly due to the rarity of these isolates in IPD. The prevalence of NTPn as a cause of IPD has been reported to range from 0.6 to 3 % [10, 12]. The majority (~90 %) of invasive NTPn described in the literature is Group I and is thought to be derived from Ec-*Sp* [10, 12]. In contrast, almost all Group II isolates are related to established NTPn lineages [12]. Exploring the potential changes in *cps* structure and understanding the genomic diversity of NTPn is of importance, especially because current pneumococcal conjugate vaccines (PCV) are not effective against these isolates. We aimed to describe disease and genotypically characterize NTPn causing IPD in South Africa.

## Results

### Invasive pneumococcal disease surveillance in South Africa, 2003–2013

From 2003 through 2013, 46,485 cases of IPD were reported, of which 32,824 (71 %) had viable isolates. Thirty-nine (0.1 %) were nontypeable by the Quellung reaction and were thus classified as NTPn. Case characteristics are shown in Table 1. Forty-four percent (17/38) of cases were in children <5 years. Two patients with known vaccination status had received one dose of PCV13 and two doses of PCV7, respectively. Thirteen percent (2/15) of patients with known immune status were immunocomprised. Of ten patients with known HIV status, 50 % (5/10) were seropositive. Twenty-two percent (4/18) of patients with known outcome data died.

**Table 1:** Characteristics of patients with invasive disease due to nontypeable and encapsulated *Streptococcus pneumoniae*, South Africa, 2003–2013

Characteristic		Nontypeable *S. pneumoniae*	Encapsulated *S. pneumoniae*	Total
		*N* = 39	*N* = 32785	*N* = 32824
		n (%)	n (%)	n (%)
Year	2003	2 (5)	2885 (9)	2887 (9)
	2004	4 (10)	3465 (11)	3469 (11)
	2005	5 (13)	3643 (11)	3648 (11)
	2006	5 (13)	3411 (10)	3416 (10)
	2007	2 (5)	3318 (10)	3320 (10)
	2008	6 (15)	3319 (10)	3325 (10)
	2009	7 (18)	3377 (10)	3384 (10)
	2010	3 (8)	2869 (9)	2872 (9)
	2011	0	2409 (7)	2409 (7)
	2012	3 (8)	2158 (7)	2161 (7)
	2013	2 (5)	1931 (6)	1933 (6)
Gender	Male	23 (59)	15842 (48)	15865 (48)
	Female	16 (41)	16291 (50)	16307 (50)
	Unknown	0	652 (2)	652 (2)
Age group (years)	<5	17 (44)	9446 (29)	9463 (29)
	5–14	3 (8)	2960 (9)	2963 (9)
	15–24	2 (5)	1918 (6)	1920 (6)
	25–44	10 (26)	11714 (36)	11724 (36)
	45–64	4 (10)	4355 (13)	4359 (13)
	>64	2 (5)	1137 (3)	1139 (3)
	Unknown	1 (3)	1255 (4)	1256 (4)
Specimen type	Cerebrospinal fluid	11 (28)	11486 (35)	11497 (35)
	Blood	22 (56)	19284 (59)	19306 (59)
	Other^a^	6 (16)	2015 (6)	2021 (6)
PCV vaccination^b^	Yes^c^	2 (100)	580 (77)	582 (77)
	No	0	131 (17)	131 (17)
	Unknown	0	46 (6)	46 (6)
PCV13 serotypes^d^	PCV13 serotypes	13^e^ (59)	23619 (72)	23632 (72)
	Non PCV13 serotypes	9^e^ (41)	9166 (28)	9175 (28)
HIV status	Positive	5 (13)	7718 (23)	7723 (23)
	Negative	5 (13)	2489 (8)	2494 (8)
	Unknown	29 (74)	22578 (69)	22607 (69)
Immuno compromising conditions^f^	Yes	2 (5)	3114 (10)	3116 (10)
	No	13 (33)	6974 (21)	6987 (21)
	Unknown	24 (62)	22697 (69)	22721 (69)
Comorbid conditions^g^	Yes	2 (5)	1496 (5)	1498 (5)
	No	13 (33)	8592 (26)	8605 (26)
	Unknown	24 (62)	22697 (69)	22721 (69)
Outcome	Died	4 (10)	4024 (12)	4028 (12)
	Recovered	14 (36)	9887 (30)	9901 (30)
	Unknown	21 (54)	18874 (58)	18895 (58)

^a^Other specimen types: pleural, joint and vitreous fluid

^b^Only children born after February 2009 were included

^c^Received at least 1 dose of the vaccine

^d^PCV13 serotypes: 4, 6B, 9V, 14, 18C, 19F, 23F, 1, 5, 7F, 3, 6A and 19A

^e^Nontypeable predicted ancestral serotypes for Group I isolates

^f^Immunocompromising conditions were defined as medical record-documented pre-existing history of head injury, connective tissue disease, asplenia, pregnancy, premature birth, malignancy, burns, gastric acid suppression, aplastic anemia, organ transplant, primary immunodeficiency conditions, chromosomal conditions, protein energy malnutrition, alcohol dependency, current smoking, or immunosuppressive therapy

^g^Comorbid conditions were defined as medical record-documented pre-existing history of pulmonary disease, renal disease, cerebrovascular accident, hepatic disease, cardiac disease, or diabetes mellitus

### Classification of NTPn and multi-locus sequence type

Fifty-six percent (22/39) of NTPn had at least partial *cps* genes and were thus categorized as Group I (Table 2). The remaining isolates (17/39, 44 %) had complete deletion of *cps* genes (*n* = 17) and replacement by *pspK* (*n* = 1), *aliC*, *aliD* and *ntaAB* (*n* = 12) or *aliC* and *aliD* genes only (*n* = 2) and were thus categorized as Group II. Based on the classification proposed by Park et al. [12, 17], these isolates were defined as NCC1 (*n* = 1), NCC2a (*n* = 12), NCC2b (*n* = 2) and NCC4 (*n* = 2).

**Table 2:**
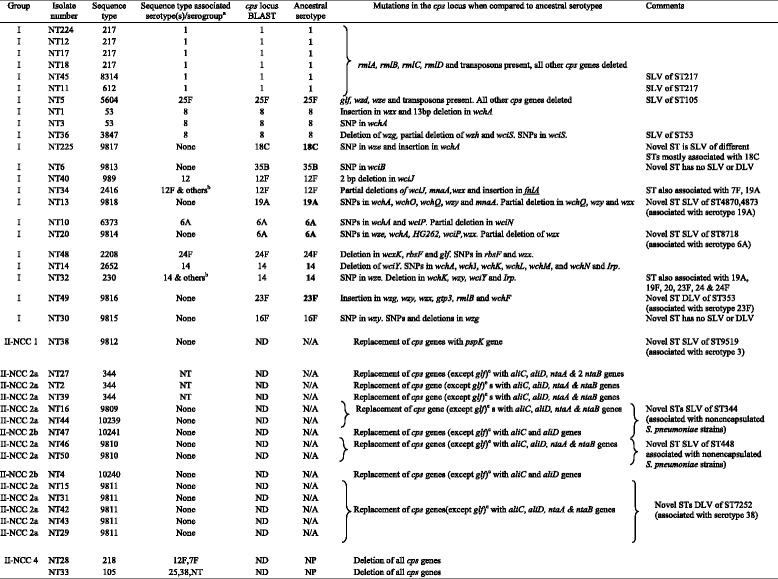
Classification, predicted ancestral serotypes and *cps* mutations of invasive nontypeable *Streptococcus pneumoniae* in South Africa, 2003–2013 (*N* = 39)

Bold type represents serotypes included in PCV13

*SLV* single-locus variant, differs at one of the seven alleles. *DLV* Double-locus variant, differs at two of the seven alleles

None Novel ST and therefore no serotype/ST association exists

*ND* not done, isolates have no *cps* genes

*N/A* Not applicable, resembles nontypeable pneumococci from carriage (no *cps* genes, but instead have typical nontypeable genes)

*NP* not predicted. Isolates have no *cps* genes and/or are associated with more than 1 serotype

^a^As per *Streptococcus pneumoniae* multilocus sequence typing database (http://pubmlst.org/spneumoniae/, accessed March 2015)

^b^Other serotype/s associated with that ST. See comments

^c^ *glf* pseudo gene present within the *cps* locus

**Fig. 1:**
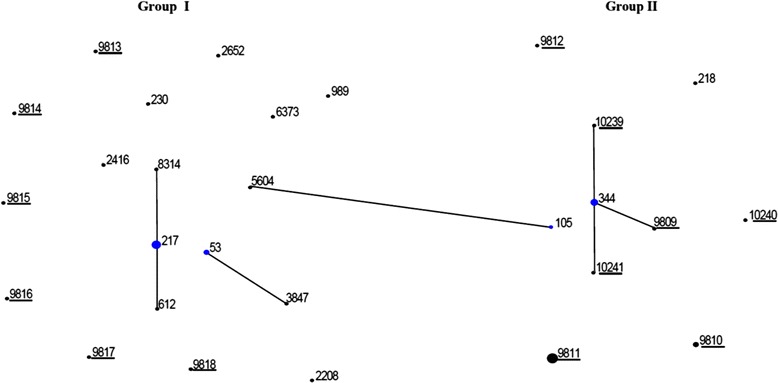
eBURST population snapshot showing relationships among sequence types (ST) identified in Group I (*n* = 22) and Group II (*n* = 17) invasive nontypeable *Streptococcus pneumoniae* in South Africa (2003–2013). The size of each circle corresponds with the number of isolates. Clusters of linked isolates correspond to clonal complexes (isolates sharing six of seven alleles) and blue indicates the founding genotype. New STs are underlined

A total of 28 STs were identified (Table 2). Among Group I isolates, 18 STs were identified: 6 were novel STs and 12 were STs usually associated with Ec-*Sp*. Among Group II isolates, 10 STs were identified: 7 were novel STs and 3 (NCC2a) were ST344 which is usually associated with carriage NTPn, and ST105 and ST218 (NCC4) which are associated with Ec-*Sp*. Two CCs were identified among Group I isolates; CC217 (*n* = 6) and CC53 (*n* = 3) (Fig. 1). The remainder (*n* = 12) of Group I isolates were not assigned to any CC, except for ST5604 which is a single-locus variant of a ST105 isolate from Group II. CC344 (*n* = 6) was identified among Group II isolates. The remainder (*n* = 10) of Group II isolates were not assigned to any CC.

### Predicted ancestral serotypes of Group I isolates

The most common predicted ancestral serotypes were serotype 1 (*n* = 6), 8 (*n* = 3), 12F (*n* = 2), 6A (*n* = 2) and 14 (*n* = 2). Fifty-nine percent (13/22) of the predicted ancestral serotypes were PCV13 serotypes 1 (*n* = 6), 18C, 19A, 6A (*n* = 2), 14 (*n* = 2) and 23F (Table 2). Two of the NTPn isolates (NT224 and NT225) were detected in two patients that were co-infected with a serotype 1 and serotype 18C isolate, respectively.

### *cps* mutations in Group I isolates

Comparisons between Group I isolates and their ancestral serotype *cps* regions revealed a variety of mutations within the *cps* region of Group I isolates. These mutations are summarized in Table 2, described in detail in Additional file 1 and sketched in Additional file 2. Briefly, predicted ancestral serotype 1 isolates had identical partial deletions of *cps* genes (NT11, NT12, NT17, NT18, NT45 and NT224). Isolate NT5 also had partial deletions. The remaining isolates had almost all *cps* genes present, as in their ancestral serotypes; however, we identified a variety of mutations [single nucleotide polymorphisms (SNPs), insertions and deletions] in some of the *cps* genes. Most isolates had a combination of mutations. The vast majority of SNPs resulted in amino acid changes and a small number in stop codons. Base deletions and insertions were not common as SNPs.

### Phylogenomic analysis

**Fig. 2:**
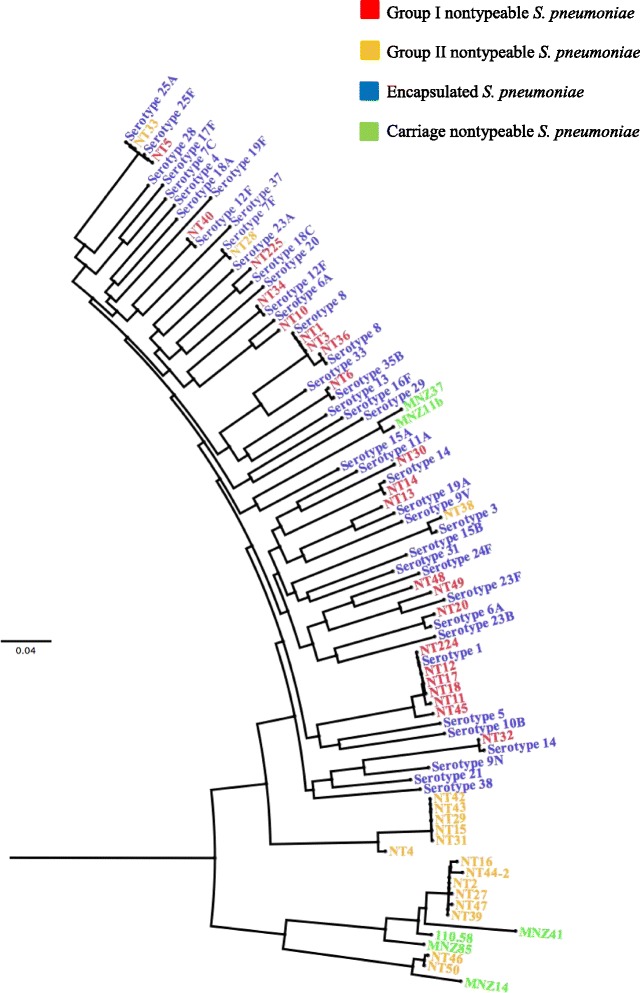
Maximum likelihood phylogenetic tree based on core genome SNPs of invasive nontypeable *Streptococcus pneumoniae* (*n* = 39), shown in red and yellow from South Africa (2003–2013). Encapsulated *S. pneumoniae* (representative of predicted ancestral serotypes) from South Africa (*n* = 42, shown in blue) and publically available nonencapsulated *S. pneumoniae* genomes ([20, 21] (*n* = 6, indicated green) were included for comparison

Ancestral serotype prediction was further confirmed by phylogenomic clustering of Group I isolates with representative genomes of their predicted ancestral serotypes (Fig. 2). An exception was NT30 which was predicted to be derived from serotype 16F but was more closely related to serotype 11A. For some isolates, there was more divergence between the NTPn and their ancestral serotypes as indicated by the long branches. Group II NCC1 (NT38), NCC4 (NT28 and NT33) and two carriage NTPn (MNZ37 and MNZ11b) from other studies [20] also clustered with invasive Ec-*Sp* and appear to be related to serotypes 3, 7F, 25A and 15A, respectively. Group II NCC2 NTPn belonging to ST9811 (*n* = 5) and ST10240 formed a distinct clade separate from other Group II NCC2 isolates and seem to be more closely related to invasive Ec-*Sp*. The remaining Group II NCC2 NTPn (*n* = 8) formed a distinct clade with carriage NTPn from other studies (*n* = 4) [20, 21]. Overall, 31/39 (79 %) of NTPn from this study ([22] Group I, Group II NCC1 (*n* = 1), NCC4 (*n* = 2) and six NCC2 isolates belonging to ST9811 (*n* = 5) and ST10240) appear to have been derived from Ec-*Sp*.

### Antimicrobial resistance patterns

**Fig. 3:**
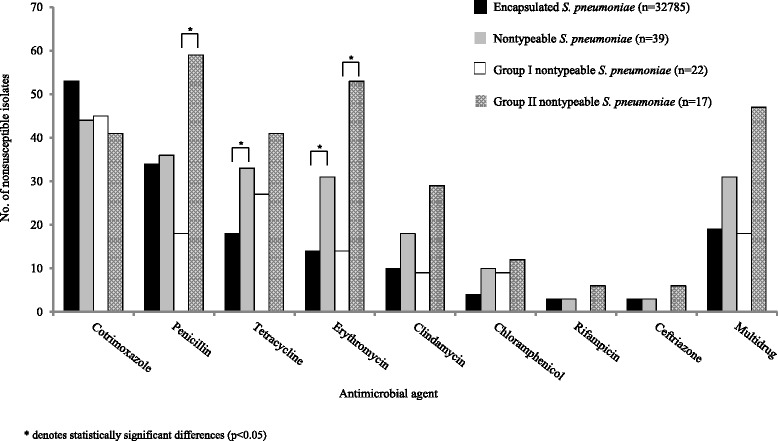
Antimicrobial nonsusceptibility of *Streptococcus pneumoniae* isolates causing invasive disease in South Africa, 2003–2013

Nonsusceptibility among NTPn was highest for cotrimoxazole (17/39, 44 %), penicillin (14/39, 36 %), tetracycline (13/39, 33 %) and erythromycin (12/39, 31 %) (Fig. 3). Prevalence of nonsusceptibility was higher among NTPn than Ec-*Sp* for most antimicrobials; however this difference was only significant for tetracycline [33 % (13/39) versus 18 %, (5902/32785), *P* = 0.02] and erythromycin [31 % (12/39) versus 14 % (4589/32785), *P* = 0.005]. In addition, 31 % (12/39) of NTPn were multidrug resistant [of which 21 % (8/39) included penicillin] compared to 19 % (6230/32785) of Ec-*Sp* however this difference was not statistically significant (*P* = 0.09). With the exception of cotrimoxazole and chloramphenicol, Group II NTPn had a higher prevalence of nonsusceptibility than Group I, although this difference was only statistically significant for penicillin [59 % (10/17) versus 18 % (4/22), *P* = 0.02] and erythromycin [53 % (9/17) versus 14 % (3/22), *P* = 0.01]. Multidrug resistance was also higher in Group II NTPn (47 %, 8/17) compared to Group I (18 %, 4/22), although not statistically significant (*P* = 0.08).

## Discussion

We characterized NTPn causing IPD in South Africa, a middle-income, high HIV prevalence country with PCV introduction in 2009. NTPn represented 0.1 % of IPD cases, much lower than other studies which have reported a prevalence of between 0.6 and 3 % [10, 12]. Our study showed that among patients with NTPn infection and for whom data were available, 44 % were children less than 5 years, proportionately more than Ec-*Sp* (29 %), 50 % were HIV positive versus 76 % for Ec-*Sp* and the case-fatality ratio (22 %) was similar to cases with Ec-*Sp* (29 %). Fifty-six percent of our NTPn were Group I, for which the most common predicted ancestral serotypes were 1 (27 %) and 8 (14 %). Fifty-nine percent of the predicted ancestral serotypes were PCV13 serotypes. The *cps* loci of the Group I isolates harbored a variety of mutations. Nonsusceptibility to tetracycline and erythromycin was significantly higher in NTPn than encapsulated *S. pneumoniae*. Nonsusceptibility to penicillin and erythromycin was also significantly higher in Group II NTPn than Group I.

The mechanisms by which NTPn are able to cause IPD without the capsule are not clear. Immune suppression of the host and/or other lesser known or novel virulence factors might possibly explain the success of NTPn in causing IPD. However, in this study the majority of patients with NTPn infection (87 %, 13/15) were not immunocompromised and this was similar to patients with Ec-*Sp* strains (69 %). NTPn appear to be equally as virulent as Ec-*Sp* as the patients with NTPn infection did not seem to be more susceptible to infection.

We observed a higher prevalence (44 %) of Group II isolates amongst our NTPn that has not been described in other studies, where 4 to 10 % of NTPn were Group II isolates [10, 12]. The majority of Group II isolates (82 %) were NCC2 and no isolates belonging to NCC3 were identified, a finding similar to invasive NTPn collected through the Active Bacterial Core surveillance program in the United States during 2006–2009 [12]. In addition, two isolates were classified as NCC4, a newly defined clade in Group II, characterized by deletion of all *cps* genes [12]. Similar to what has been shown in the US Active Bacterial Core surveillance, NCC4 isolates in this study also appeared to be derived from Ec-*Sp* [12].

We were able to predict ancestral serotypes for all Group I isolates because they had partial *cps* genes, suggesting that these isolates were derived from Ec-*Sp* which, at some point, lost their capsule through mutations in the *cps* locus. Indeed we found a diverse range of mutations in the *cps* locus of Group I isolates, some of which may be responsible for the nontypeable phenotype. For predicted ancestral serotype 1 isolates, all *cps* mutations were identical. The deleted region is flanked by identical IS1167 repeats and this, together with the fact that the predicted ancestral serotype 1 isolates were genotypically related, indicates that the *cps* mutations probably occurred as a result of a single deletion event in a particular strain rather than multiple independent deletions in different strains. The same mutations have been described for serotype 1-derived NTPn in other studies [9, 10].

Serotype predictions were confirmed by the phylogenomic analyses as these isolates clustered with encapsulated strains expressing the same serotype as the predicted ancestral serotypes. One isolate (NT30), however, was more closely related to serotype 11A than its predicted serotype 16F. This isolate has a novel ST, which shares three of seven alleles with the 11A isolate, suggesting that NT30 may have been derived from a common ancestor with serotype 11A at some point in time. For some isolates, there was more divergence between the NTPn and their ancestral serotypes. This could be that certain serotypes are inherently diverse while others are more stable.

Predicted ancestral serotypes 1 (27 %) and 8 (14 %) were most prevalent among our NTPn isolates. Analysis of invasive NTPn isolates from Native American communities collected from 1994–2007 showed that predicted ancestral serotype 1 (19 %) and 7 F (15 %) were most prevalent [10]. In addition, serotype 8 (25 %) was most prevalent among invasive NTPn from the US Active Bacterial Core surveillance [12]. These data suggest that serotypes 1 and 8 may be more prone to mutations in their *cps* region than other serotypes.

Two of the NTPn isolates (NT224 and NT225) were detected in mixed infections in two patients with encapsulated serotype 1 and 18C isolates, respectively. For the serotype 1 and NT224 mixed infection, *S. pneumoniae* was identified from the same culture, with the NT isolate representing approximately 98 % of the culture (using the Quellung reaction). After several attempts to separate the two variants, only the NTPn isolate could be obtained, however, real-time PCR confirmed the presence of the serotype 1 in the mixed culture. NT224 was confirmed to be ST217 which is associated with serotype 1. For the serotype 18C and NT225 mixed infection, both isolates were available and NT225 had the same *cps* region, with the exception of a single SNP, compared to its encapsulated counterpart (serotype 18C), the same ST and their core genomes were identical (unpublished observations).

Pneumococcal serotypes targeted by PCV have the potential to switch from vaccine serotype to non-vaccine serotype or to nontypeable, thereby providing a mechanism whereby they may be able to evade vaccine pressure. However, no evidence of adaptation to PCV occurred among invasive NTPn from Native American communities, where 45 % of NTPn isolates had ancestral PCV7 serotypes pre-vaccine and none of their NTPn isolates were ancestral PCV7 serotypes post-vaccine [10]. Although 59 % of the predicted ancestral serotypes of NTPn in our study were PCV13 serotypes, there is no evidence to suggest that serotype switching occurred as a result of PCV pressure as the majority of NTPn were already present prior to PCV introduction.

Antimicrobial nonsusceptibility data for invasive NTPn are limited. In our study, there was a significantly higher prevalence of nonsusceptibility among NTPn than Ec-*Sp* for erythromycin and tetracycline. These higher nonsusceptibility rates coupled with higher transformation rates of NTPn [16] enable these strains to serve as a reservoir of antibiotic resistance genes. A serotype 19F clone from Switzerland became increasingly resistant to penicillin by acquisition of a *pbp*2x gene from NTPn [23]. Recently, a comparative genome analysis of over 3000 carriage pneumococci from a refugee camp in Thailand showed that the highest rates of receipt and donation of recombinant of DNA occurred in NTPn and that the most commonly exchanged genes were those associated with antibiotic resistance and immune interactions [24].

It is possible that our NTPn isolates may have lost the ability to express capsule during sub-culturing in the laboratory, but we believe this was not the case as we would expect NTPn to be detected more frequently. We did not perform in vitro experiments to confirm whether the mutations identified in the *cps* locus of our isolates were responsible for the lack of capsule expression. However, in some of these isolates, there were complete deletions of *cps* genes making expression of a capsule impossible. For Ec-*Sp* isolates different methods [agar dilution or Etest (2003–2008) or broth microdilution (2009–2013)] were used for MIC testing compared to NTPn (broth microdilution). This may have overestimated the prevalence of β-lactam resistance in NTPn for the 2003–2008 period as broth microdilution detects more resistance than agar dilution as was previously shown [25].

## Conclusion

NTPn are currently a rare cause of IPD in South Africa and are genetically diverse but have a higher prevalence of antimicrobial resistance than Ec-*Sp*. The majority of NTPn were derived from Ec-*Sp*, of which a significant proportion was PCV13 serotypes. Further studies which explore capsule-independent virulence mechanisms of NTPn should be considered.

## Methods

### Bacterial isolates

Isolates were obtained through the GERMS-SA (Group for Enteric, Respiratory and Meningeal Disease Surveillance in South Africa) network, which conducts active, national, laboratory-based surveillance for IPD. Surveillance began in 1999 and was enhanced in 2003 [26, 27]. Over 200 diagnostic laboratories across South Africa routinely submit clinical isolates and basic patient demographic data to the network. A case of IPD was defined as the detection of *S. pneumoniae* from a normally sterile site (e.g., cerebrospinal fluid, blood) from January 2003 through December 2013. Isolates were cultured on 5 % horse blood agar (Diagnostic Media Products, Johannesburg, South Africa) and incubated at 37 °C in 5 % CO_2_ for 18–24 h. Optochin sensitivity and bile solubility assays were performed to confirm *S. pneumoniae* identification. In addition, for NTPn isolates, real-time PCR detecting *lytA* was performed [28]. A mixed infection was defined as simultaneous isolation of at least two serotypes (including nontypeable isolates) either from the same normally-sterile site specimen or from two or more normally-sterile site specimens obtained from the same patient within 21 days of each other. Such cases were detected during routine serotyping by Quellung if an isolate reacted partially with a specific antiserum pool.

### Serotyping and antimicrobial susceptibility testing

Serotyping was performed by the Quellung reaction using serotype-specific antisera (Statens Serum Institut, Copenhagen, Denmark). Minimum inhibitory concentrations (MIC) for NTPn isolates were determined by broth microdilution using commercially customized TREK panels (Trek Diagnostics Inc., Ohio, United States). MICs for Ec-*Sp* isolates were determined as previously described [25, 27]. MIC breakpoints were interpreted using Clinical and Laboratory Standards Institute guidelines [29]. For penicillin, isolates with MICs of ≥0.12 mg/L were defined as nonsusceptible. Isolates that were intermediately resistant or resistant to any of the antibiotics were regarded as nonsusceptible. Multidrug resistance was defined as nonsusceptibility to three or more classes of antibiotics.

### DNA preparations, genome sequencing and assembly

Brain heart infusion broth (Diagnostic Media Products) was inoculated with overnight cultures and incubated overnight at 37 °C in 5 % CO_2_. A pre-lysis step was performed by suspending bacterial pellets in 200 μl of 10 mg/ml lysozyme (Sigma-Aldrich, St. Louis, MO, USA) and incubating at 37 °C for 1 h. Genomic DNA was extracted using the QIAamp® DNA Mini kit (QIAGEN, Hilden, Germany). Paired-end libraries (2 x 300 bp) were prepared using the Nextera XT DNA sample preparation kit (Illumina, San Diego, CA, USA) and sequencing was performed on an Illumina MiSeq. The resulting paired-end reads were quality trimmed and mapped to the reference genome of *S. pneumoniae* ATCC 700669 [GenBank accession no. FM211187, serotype 23 F, sequence type (ST) 81] using CLC Genomics Workbench v8 (CLC bio, Aarhus, Denmark), giving on average 121x depth of coverage and 88 % coverage of the reference genome. *De novo* assembly was performed for all isolates using CLC Genomics Workbench and ordered relative to *S. pneumoniae* ATCC 700669 using Mauve [30]. The ordered contigs were concatenated and annotated using Prokka v1.11 [31].

### *cps* regions and multi-locus sequence typing (MLST)

The *cps* locus and seven MLST alleles were derived from whole genome data. The *cps* loci were used to categorize NTPn into different groups/clades [12, 17]. MLST allele numbers and sequence type (ST) were assigned using the Bio-MLST-Check module (http://search.cpan.org/~ajpage/Bio-MLST-Check 1.133090/lib/Bio/MLST/Check.pm). The eBURST v3 algorithm (http://eburst.mlst.net) was used to determine genetic relationships and grouped isolates into clonal complexes (CC) [32]. A CC was defined as a group of isolates sharing six of seven alleles with any other isolate in the group.

### *In silico* prediction of ancestral serotypes for Group I NTPn

To predict ancestral serotypes of Group I isolates (serotypes from which NTPn may have originated), two methods were used, namely, serotype-ST associations using the MLST database (http://pubmlst.org/spneumoniae), accessed March 2015, and blasting of the *cps* locus against reference *cps* loci for the 94 known serotypes (GenBank accession no’s. CR93162 – CR931722, EF538714, HM171374, GU074953 and JQ653094). The serotype that gave the highest BLAST bit score was assigned as the likely ancestral serotype.

### *cps* mutations in Group I NTPn

To determine the genetic variations within the *cps* locus of Group I isolates that may be responsible for their phenotypic nontypeability, we used the alignment module in CLC to align the *cps* locus of each isolate together with a number of its predicted ancestral serotype *cps* loci shown in Additional file 3.

### Phylogenomic tree construction

To confirm predicted ancestral serotypes for Group I isolates, a maximum likelihood phylogenetic tree was constructed based on core genome SNPs of NTPn from this study (*n* = 39), together with invasive Ec-*Sp* with different serotypes from South Africa (*n* = 42) Additional file 4 matching the predicted ancestral serotypes for the NTPn isolates, and carriage NTPn from other countries (*n* = 6) [20, 21]. The core genome alignment module in the rapid large-scale prokaryote pan genome analysis pipeline was used to extract predicted coding regions from annotated assemblies and convert them to protein sequences [33]. All protein sequences were compared with each other using BLASTP. Proteins that had alignment similarity of ≥70 % and were present in at least 90 % of the isolates were defined as the core genome. RAxML was used to create a phylogenetic tree from the resulting core genome alignment and this was visualized in Figtree v1.4.2 [34].

### Statistical analysis

Differences between NTPn versus Ec-Sp antimicrobial nonsusceptibility and between Group I and Group II NTPn were analyzed using the chi-square test and Fisher’s exact test, respectively, with statistical significance assessed at *P* < 0.05. Statistical analyses were performed with GraphPad InStat 3.

## Abbreviations

CC, clonal complex; *cps*, capsular polysaccharide synthesis locus; Ec-*Sp*, encapsulated *Streptococcus pneumoniae*; GERMS-SA, Group for Enteric, Respiratory and Meningeal Disease Surveillance in South Africa; IPD, invasive pneumococcal disease; MIC, Minimum inhibitory concentration; MLST, multi locus sequence typing; NCC, null capsule clade; NTPn, nontypeable pneumococci; PCV, pneumococcal conjugate vaccine; SNP, single nucleotide polymorphism; ST, sequence type.

## Additional files

Additional file 1: Description of the *cps* mutations of nontypeable *Streptococcus pneumoniae* (*n* = 39) from South Africa, 2003–2013. (XLS 44 kb) Additional file 2: Diagrammatic representation of capsular locus of nontypeable *Streptococcus pneumoniae* (*n* = 39) from South Africa, 2003–2013. (DOCX 1547 kb) Additional file 3: Accession numbers for encapsulated *S. pneumoniae* used for *cps* locus alignment (XLS 54 kb) Additional file 4: Accession numbers for encapsulated *S. pneumoniae* isolates from South Africa and carriage nontypeable isolates from other studies used to construct the phylogenetic tree. (XLS 34 kb)
