# Supplementary material for: Genomic analysis of nontypeable pneumococci causing invasive pneumococcal disease in South Africa, 2003–2013
Source: BMC Genomics. 2016 Jun 22;17:470. doi: 10.1186/s12864-016-2808-x (PMC4928513; doi:10.1186/s12864-016-2808-x)
Supplement: Additional file 2: — Diagrammatic representation of capsular locus of nontypeable Streptococcus pneumoniae (n = 39) from South Africa, 2003–2013. (DOCX 1547 kb) [file 12864_2016_2808_MOESM2_ESM.docx]

1. Serotype 1 reference strain and NT11, NT12, NT17, NT18, NT45 NT224

Serotype 1


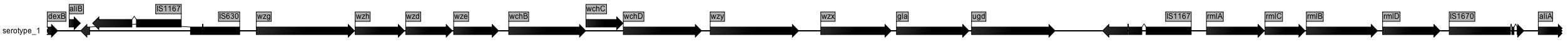

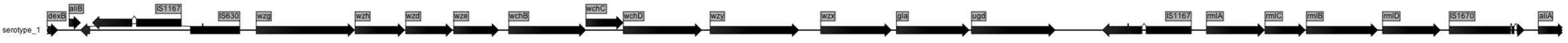

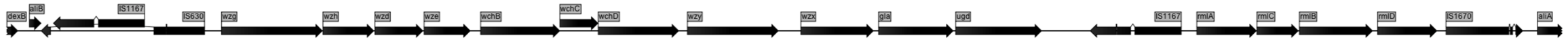


NT11, NT12, NT17, NT18, NT45 and NT224

1. Serotype 25F reference strain and NT5

Serotype 25F


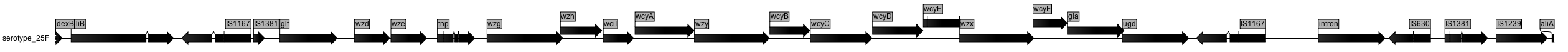

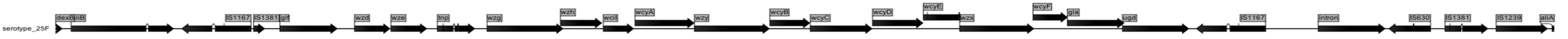

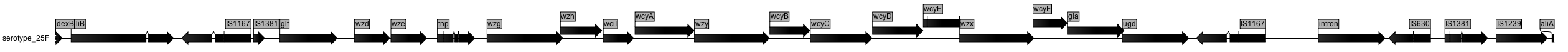

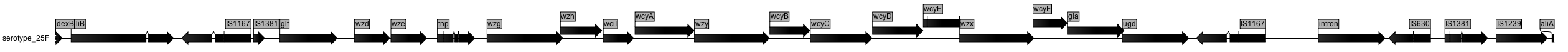

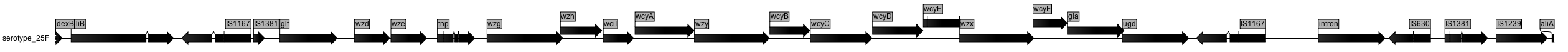

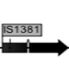


NT5

1. NT1


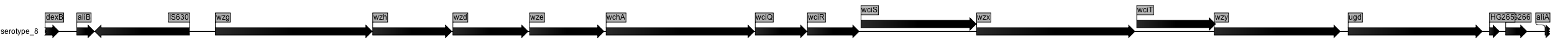


1. NT3


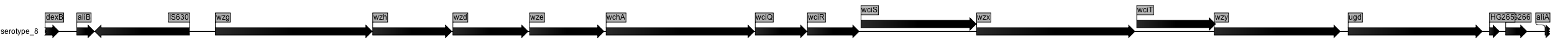


1. Serotype 8 reference strain and NT36

Serotype 8


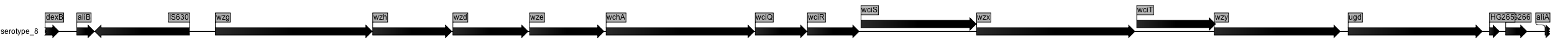

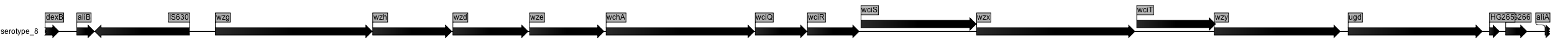

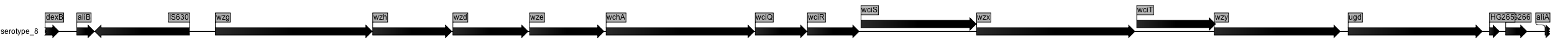


NT36

1. NT225


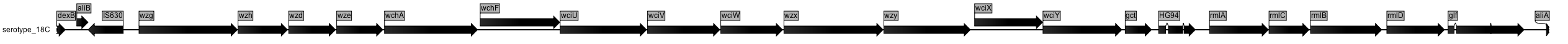


1. NT6


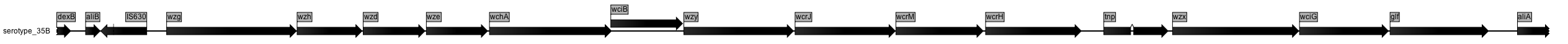


1. NT40


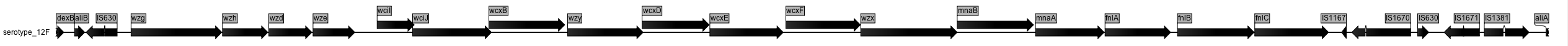


1. NT34


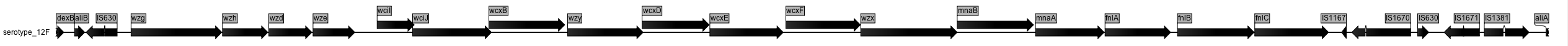


1. NT13


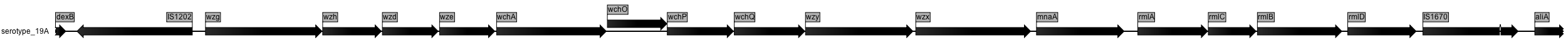


1. NT10


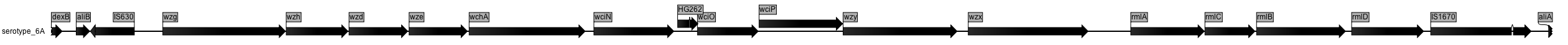


1. NT20


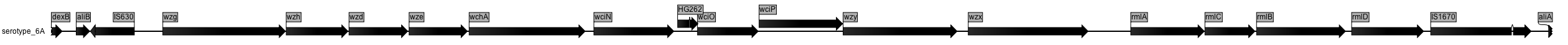

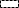

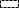


1. NT48


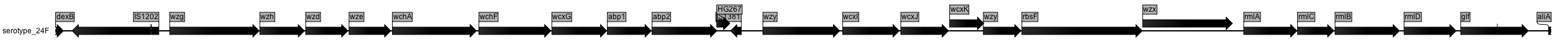


1. NT14


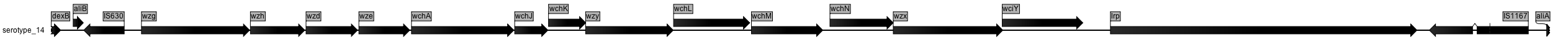

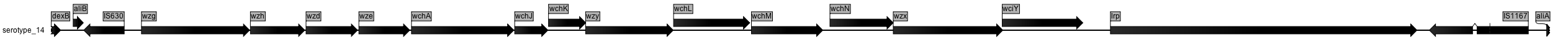


1. NT32


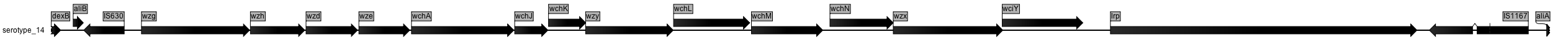


1. NT49


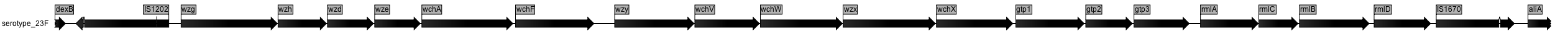


1. NT30


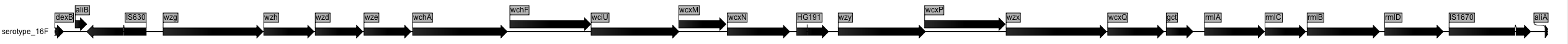


1. NT38


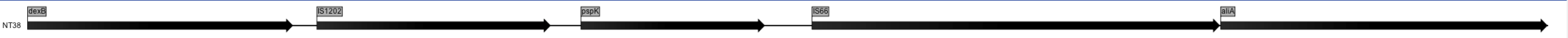


1.
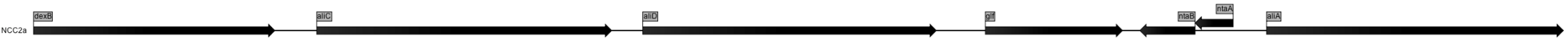
NT2, NT15, NT16, NT29, NT31, NT39, NT42, NT43, NT44, NT46 and NT50


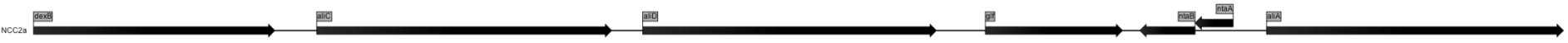


1. NT4, NT47


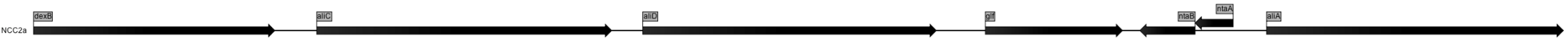

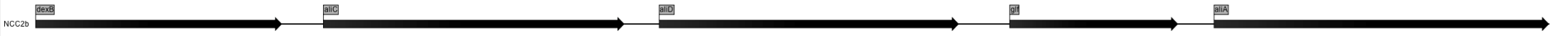


1. NT27


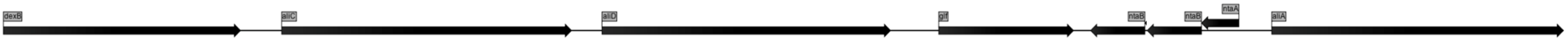


1. NT28


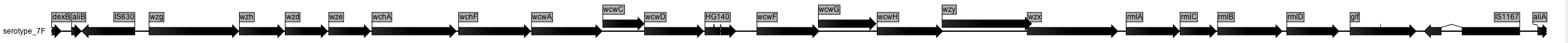

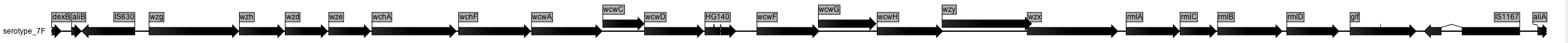


1. NT33


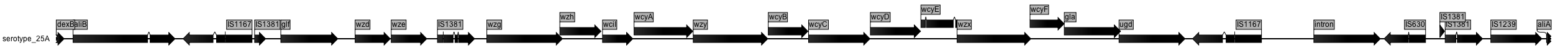

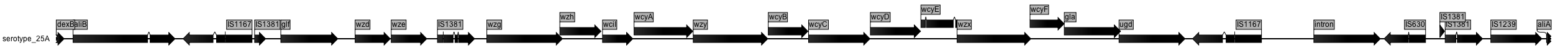


**Additional file 4**. Diagrammatic representation of the capsular locus of nontypeable *Streptococcus pneumoniae* (n=39) from South Africa, 2003-2013. Nucleotide identity is indicated by grey boxes; single nucleotide polymorphisms by horizontal lines; insertions by down arrows and deletions by white boxes with dashed lines. A detailed description of the mutations (position, nucleotide and amino acid changes) is shown in additional file 3. (a) Isolates NT11, NT12, NT17, NT18, NT45 and NT224 (bottom) were compared to their predicted ancestral serotype 1 (top) (b) NT5 (bottom) was compared to its predicted ancestral serotype 25F (top) (c) NT1 (d) NT3 (e) NT36 (bottom) was compared to its ancestral serotype 8 (top) (f) NT225 (g) NT6 (h) NT40 (i) NT34 (j) NT13 (k) NT10 (l) NT20 (m) NT48 (n) NT14 (o) NT32 (p) NT49 (q) NT30 (r) NT38 (s)
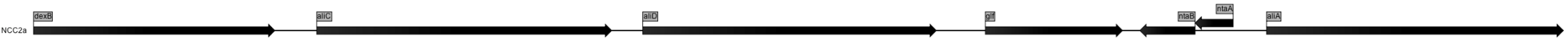
NT2, NT15, NT16, NT29, NT31, NT39, NT42, NT43, NT44, NT46 and NT50 (t) NT4 and NT47 (u) NT27 (v) NT28 (w) NT33
